# Radioimmunotherapy for Brain Metastases: The Potential for Inflammation as a Target of Choice

**DOI:** 10.3389/fonc.2021.714514

**Published:** 2021-08-24

**Authors:** Aurélien Corroyer-Dulmont, Cyril Jaudet, Anne-Marie Frelin, Jade Fantin, Kathleen Weyts, Katherine A. Vallis, Nadia Falzone, Nicola R. Sibson, Michel Chérel, Françoise Kraeber-Bodéré, Alain Batalla, Stéphane Bardet, Myriam Bernaudin, Samuel Valable

**Affiliations:** ^1^ Medical Physics Department, CLCC François Baclesse, Caen, France; ^2^ Normandie Univ, UNICAEN, CEA, CNRS, ISTCT/CERVOxy Group, GIP CYCERON, Caen, France; ^3^ Grand accélérateur National d’Ions Lourds (GANIL), CEA/DRF-CNRS/IN2P3, Caen, France; ^4^ Nuclear Medicine Department, CLCC François Baclesse, Caen, France; ^5^ Medical Research Council, Department of Oncology, Oxford Institute for Radiation Oncology, University of Oxford, Oxford, United Kingdom; ^6^ GenesisCare, Alexandria, NSW, Australia; ^7^ Team 13-Nuclear Oncology, CRCINA, INSERM, CNRS, Nantes University, Nantes, France; ^8^ Nuclear Medicine Department, University Hospital, Nantes, France

**Keywords:** brain metastases, radio-immunotherapy, microenvironment, alpha-particle therapy, inflammation, VCAM-1, 212Pb

## Abstract

Brain metastases (BM) are frequently detected during the follow-up of patients with malignant tumors, particularly in those with advanced disease. Despite a major progress in systemic anti-cancer treatments, the average overall survival of these patients remains limited (6 months from diagnosis). Also, cognitive decline is regularly reported especially in patients treated with whole brain external beam radiotherapy (WBRT), due to the absorbed radiation dose in healthy brain tissue. New targeted therapies, for an earlier and/or more specific treatment of the tumor and its microenvironment, are needed. Radioimmunotherapy (RIT), a combination of a radionuclide to a specific antibody, appears to be a promising tool. Inflammation, which is involved in multiple steps, including the early phase, of BM development is attractive as a relevant target for RIT. This review will focus on the (1) early biomarkers of inflammation in BM pertinent for RIT, (2) state of the art studies on RIT for BM, and (3) the importance of dosimetry to RIT in BM. These two last points will be addressed in comparison to the conventional EBRT treatment, particularly with respect to the balance between tumor control and healthy tissue complications. Finally, because new diagnostic imaging techniques show a potential for the detection of BM at an early stage of the disease, we focus particularly on this therapeutic window.

## Introduction

### Current Management of Brain Metastases

Owing to advances in primary cancer control, the incidence of brain metastases (BM) is increasing (1). Lung cancer, the main cause of death from cancer, and breast cancer, the most common cancer in women in developed countries, carry 40% and 20% risk of BM, respectively (2). Depending on the Karnofsky performance status (KPS) factor, molecular features, and number of BM of the patient, conventional treatment comprises surgical resection when possible and image-guided stereotactic radiosurgery (SRS), with or without whole-brain radiotherapy (WBRT) (1).

### Therapeutic Challenges

Despite these treatments, and even in cases where control of the primary cancer has a favorable impact on the overall survival (OS), a significant proportion of patients die as a result of BM⁠ (3), with an average OS of 6 months. For WBRT, 30 Gy in 10 fractions is conventionally given which can lead to cognitive decline owing to the radiation absorbed dose in healthy brain tissue. Indeed, cognitive changes were observed in children after a WBRT dose of greater or equal to 18 Gy. However the effect of WBRT in the adult brain is less well defined, with the incidence and severity of cognitive decline dependent on the dose per fraction, fractionation frequency, and volume irradiated (4). Therefore, increasing the dose of external radiotherapy (RT) in an effort to improve tumour control is currently not possible.

The balance between Tumour Control Probability (TCP) and Normal Tissue Complication Probability (NTCP) are, therefore, sub-optimal with the current treatments. One explanation may be the fact that BM are often diagnosed when locally advanced, as conventional MRI only detects the late disruption of the blood-brain-barrier (BBB) (5)⁠, when tumours are large and frequently beyond effective treatment. In contrast, treatment in the earlier stages of BM development is likely to yield a better tumour control, as fewer tumour cells have invaded the brain parenchyma and those that have remain within easier reach of systemic therapies if access across the BBB can be negotiated. Thus, the pressing unmet therapeutic challenges are to treat (i) at an early stage when relatively few metastatic tumour cells have invaded the brain parenchyma, and (ii) in a targeted manner to avoid healthy brain toxicity.

Radioimmunotherapy (RIT) enables targeted dose delivery by systemically administered radiopharmaceuticals to disseminated cancer cells. RIT uses the combination of a radionuclide emitting ionizing particulate radiation coupled to an antibody that targets a specific antigen expressed on tumour cells or their local microenvironment. For this reason, unlike conventional RT, RIT specifically affects cells that express the relevant molecular target (6)⁠, limiting dose deposition in healthy tissues, even those close to the tumour mass. The potential of RIT has been shown, for example, in non-Hoddgkin’s lymphoma where ^131^I was combined with an anti-CD20 antibody (7).

However, to the best of our knowledge, only one clinical study with four patients has evaluated the therapeutic relevance of RIT in BM. Poli and colleagues used a fully humanized antibody L19, which targets an epitope contained in the extra-domain B (EDB) of fibronectin (8)⁠. EDB-containing fibronectin molecules are highly expressed in the extracellular matrix surrounding newly formed blood vessels. Since most solid tumours and hematologic malignancies rely on neoangiogenesis for their growth and metastatic spread, it makes EDB-containing fibronectin an ideal target for RIT. ^131^I-L19SIP (Radretumab) administration resulted in a decrease in tumour glucose metabolism with a significant BM/background uptake ratio > 4. From these studies, the attributes of RIT make it a promising approach to early BM management and could lead to a better TCP/NTCP ratio. Given the high linear energy transfer (LET), especially for alpha particles, and local dose deposition of radiation emitting particles, the specificity of the target is crucial. To this end, exploring the biomarkers of early BM or the microenvironment of early BM development could be a first step in providing an alternative target for RIT.

## Early Biomarkers of BM

### Biomarkers of Tumour Cells

For about 30% of the patients, BM resulting from some lung and breast cancers, as well as the primary cancer cells themselves, exhibit an overexpression of the epidermal growth factor receptor (EGFR) and HER2. For this reason, tyrosine kinase inhibitors (TKI), such as ALK inhibitor, are used as systemic treatments in both the early and late disease stages and improve the progression-free survival of patients compared to chemotherapy. Thus, the combination of a TKI and RIT, in a way to transport the RIT, may have a potential as a new therapeutic strategy. However, the expression of EGFR and HER2 is highly heterogeneous, and TKI cannot be proposed for all patients (2). Another useful target for RIT could be the overexpression of certain genes that are implicated in early BM. Duchnowska and colleagues have shown, in 84 patients with breast cancer, that the expression of RAD51, HDGF, and TPR could predict early BM development and could be used as intracellular targets (9). Intracellular targets for RIT are relevant as their close proximity to DNA means that even radionuclides with very short-range emissions, such as the Auger-emitting ^125^I, become candidates for therapy. Nevertheless, these targets require the radiopharmaceutical to traverse the BBB and cancer cell plasma membrane, yielding a significant delivery challenge. Alternatively, αv-integrins which play a role in tumour cell adhesion, invasion, and growth, have also been proposed as early biomarkers of BM. In a preclinical study, administration of intetumab, an anti-αv-integrin-targeted monoclonal antibody, was shown to decrease BM formation and increase the overall survival in a breast cancer BM rat model (10). Importantly, αv-integrins are expressed on the cell membrane surface and are therefore more accessible for RIT targeting than intracellular targets.

All of these cancer cell biomarkers are promising as therapeutic targets, but to date, most systemically administrated agents have failed to provide effective treatment for BM owing to the presence of the BBB, which remains effective when addressing the early phase of BM. Puttemans and colleagues have shown that even the early phases of BM growth are characterized by a functional BBB (11). RIT agents directed against cancer cell biomarkers must be delivered efficiently so that sufficient radioactivity accumulates intratumourally to cause radiotoxicity. An alternative approach, however, is to use more accessible molecular targets, even if these are not on or immediately adjacent to cancer cells. This option is feasible because of the penetration of particulate radiation in tissue, resulting in radial radiation dose deposition from the decay site itself. Thus, RIT can be targeted to compartments relatively close to the tumour and still remain effective in treating tumour cells.

### Biomarkers of the Tumour Microenvironment

#### Vascularisation

Kienast and colleagues have demonstrated, in preclinical models, the importance of vascular remodelling at the very early stages of BM invasion into the brain parenchyma. Using a multiphoton laser scanning microscopy, these investigators demonstrated the onset of BM formation through real-time tracking of individual human melanoma and lung cancer cells that had been injected *via* the mouse heart into the circulation (12). Early BM development appears to be strongly correlated with the ability to stimulate angiogenesis. Interestingly, it was shown that anti-angiogenic treatments (e.g., anti-VEGF, bevacizumab) could decrease the establishment of BM from lung carcinoma. These findings are consistent with the ability of bevacizumab to prevent BM development from nsNSCLC (AVAiL trial), but not BM from breast cancer (AVADO and AVEREL trials) (13). Whereas angiogenesis occurs after tumour cell invasion into the brain parenchyma, endothelial cells may express markers such as connexion as a direct result of tumour cell extravasation, providing targets indicative of an even earlier stage of BM (14).

The vascular compartment appears to be a rational target for RIT of early BM and the combination with anti-angiogenic treatments, such as bevacizumab, merits investigation. However, depending on the choice of the radionuclide, radio-toxicity in healthy tissue could be an issue. Angiogenesis is not only observed within the tumour microenvironment, VEGF is also expressed in healthy endothelial cells, macrophages, and platelets, whilst VEGF also plays a role in normal physiological functions such as bone formation, haematopoiesis, and development. In the tumour microenvironment, on the other hand, radio-toxicity on the vasculature could increase vessel permeability and provide a means to improve systemic drug delivery to BM. As an example, ^225^Ac coupled to a monoclonal antibody directed against monomeric vascular endothelial cadherin, which is expressed on the tumour neovasculature (E4G10), induces vascular remodelling in a preclinical model of glioblastoma (15). This remodelling impacted the biodistribution of a systemic treatment, with an increase of dasatinib (TKI) concentration observed within the tumour when given in combination with the RIT agent. Vascular remodelling using RIT increased tumour permeability by 58% and was concomitant with an increase in the overall survival in mice from 9 to 21 days in comparison to the control. Finally, targeting fibronectin may be promising, as the molecule is present in the extracellular matrix surrounding newly formed blood vessels in BM and is undetectable in almost all healthy adult tissues (with the exception of female reproductive organs), which has a potential for healthy tissue preservation in the case of targeting RIT (8, 16).

#### Inflammation

Inflammatory processes are known to play a key role in the early invasion of the brain parenchyma by metastatic cancer cells. Leukocyte recruitment after tumour cell invasion is well characterized, and therapeutic trials using immunomodulation have yielded promising results, despite the notable heterogeneity between patients (17, 18). The endothelial cellular adhesion molecules (CAMs), which are implicated in the adhesion and transendothelial migration of macrophages and T cells, are also co-opted for tumour cell traversal through the endothelium. ALCAM, E-selectin, ICAM/LFA-1, and VCAM-1/VLA-4 have all been shown to play a part in the tumour cell invasion into the brain parenchyma (19). For this reason, these proteins have a considerable potential as biomarkers of early BM. In preclinical studies, blocking VLA-4 or ALCAM on tumour cells (*via* incubation with neutralizing antibodies) resulted in a significant decrease in the number and volume of BM in comparison to the unblocked cells (19). Importantly, endothelial CAM overexpression has been observed in early BM in both preclinical studies and human tissue (20, 21). VCAM-1, in particular, has been presented as a major biomarker of tumorigenesis in many types of cancers, further reinforcing its early biomarker status (22). On this basis, a novel MRI contrast agent, comprised of microparticles of iron oxide (MPIO) conjugated to anti-VCAM-1 antibodies (VCAM-MPIO), has been proposed as a diagnostic tool for the detection of early BM (23), and has been shown to enable detection of BM from breast, lung, and melanoma human cancers in preclinical models (24).

For these reasons, RIT targeted to BM *via* VCAM-1 may be promising, as it allows the targeting of the very early phase of BM. However, it is important to keep in mind that CAMs are also expressed in normal tissue, such as the kidney and bone marrow, and so toxicity profiles should be evaluated with care.

## Radioimmunotherapy of Early BM

RIT is the combination of a specific targeting moiety with a specific payload. In terms of payload, a range of radionuclides with different physicochemical properties can be used in RIT, each with different advantages and limitations (**Table 1**). This diversity of available radionuclides provides a means of matching treatment to the tumour characteristics. As previously discussed, early BM exhibits a functional BBB, which prevents an easy direct contact between the systemically administered RIT and tumour cells. Radionuclides can damage the tumour DNA from a distance. This distance depends on the energy and, therefore, track length in tissue, of the particulate emissions. Ranges are of the order of a few nm–µm for Auger e^-^, µm for α particles, or mm for β particles. Energy deposition also depends on the type of particles: dozen of keV for Auger e^-^, hundreds of keV for β particles, and MeV for α particles (25). Given these properties, RIT targeting of VCAM-1 expression on endothelial cells could result in the irradiation of adjacent early BM. However, which radionuclide is most suitable for this application has yet to be investigated.

**Table 1:** Early biomarkers of BM and potential radionuclides to combined.

Biomarkers (study reference)	Potential radionuclides for RIT (type of emission)	Therapeutic particle range	Radionuclide allowing biodistribution/dosimetry evaluation?	Advantages	Limitations

**RAD51, HDGF and TPR gene overexpression in primary cancer cells** (8)	^125^I, ^111^In (e-/γ)	2-5 nm	yes	•No need to pass throught the BBB	•Need to be internalized into the cells
				•Early stage of BM	•Low energy deposition
				•Possible biodistribution/dosimetry evaluation	
**Tyrosine kinase inhibitors ** (2)	^212^Pb, ^225^Ac, ^211^As, ^213^Bi (α/β-/γ)	40-100µm	no	•Early stage of BM	•Need to pass throught the BBB
					•Difficult biodistribution/dosimetry evaluation
**αv-integrin** (9)	^212^Pb, ^225^Ac, ^211^As, ^213^Bi (α/β-/γ)	40-100µm	no	•Early stage of BM	•Need to pass throught the BBB
					•Difficult biodistribution/dosimetry evaluation
**VEGF** (12)	^212^Pb, ^225^Ac, ^211^As, ^213^Bi (α/β-/γ)	40-100µm	no	•Early stage of BM	•Difficult biodistribution/dosimetry evaluation
				•No need to pass throught the BBB	•VEGF expression in healthy tissue could induce radiotoxicity
**VCAM-1** (21)	^212^Pb, ^225^Ac, ^211^As, ^213^Bi (α/β-/γ)	40-100µm	no	•Early stage of BM	•Difficult biodistribution/dosimetry evaluation
				•No need to pass throught the BBB	•VCAM-1 expression in kidney, spleen and bone marrow could induce radiotoxicity

Based on the two-photon and immunohistochemistry images from a preclinical model of breast cancer BM (MDA-231-Br cell line), an *in silico* model was constructed to evaluate several radionuclides to identify which would provide the best radiation dose distribution in the context of early BM formation. In this study, Monte Carlo simulations were performed with ^149^Tb, ^211^At, ^212^Pb, ^213^Bi, and ^225^Ac (α-emitting radionuclides); ^90^Y, ^161^Tb, and ^177^Lu (β-emitting radionuclides); and ^67^Ga, ^89^Zr, ^111^In, and ^124^I (auger e^–^emitting radionuclides) enabling evaluation of dose deposition in the DNA specifically. This study showed that α particle emitters, with a short range and a high dose deposition, are the most appropriate for RIT targeting *via* VCAM-1 expression in early BM. Among these α particles emitters, ^212^Pb has the attributes of a theranostic radionuclide since it can be used for SPECT imaging and showed a favourable dose profile and RBE (26).

**Figure 1:**
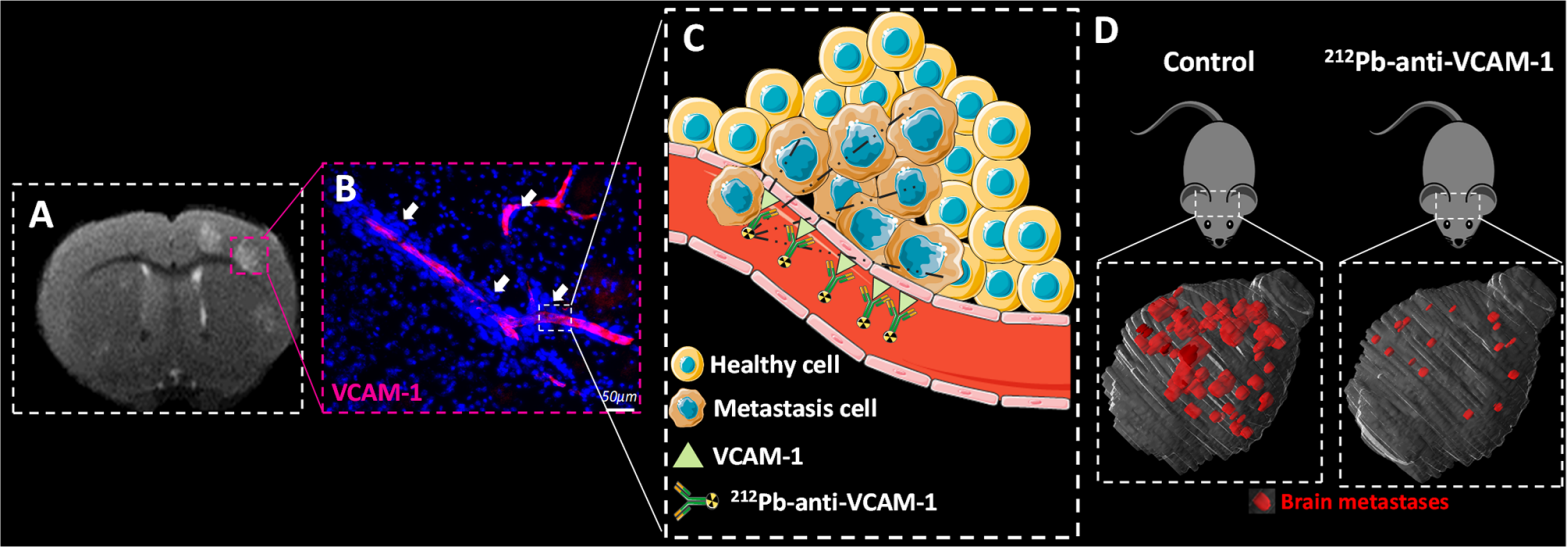
RIT in early brain metastases. **(A)** MRI of a brain mouse with early brain metastases (BM). **(B)** VCAM-1 immunostaining showing the small distance between VCAM-1 activated endothelial cells and BM (pink staining and with arrow). **(C)** Representation of radioimmunotherapy targeting VCAM-1 for the early treatment of BM. **(D)** 3D representation of BM (red) in the brain (grey) in the control and 212Pb-anti-VCAM-1 treated group.

On the basis of the above study, the therapeutic value of RIT for early BM was assessed in a preclinical study, in which the added-value of RIT using ^212^Pb combined with an anti-VCAM-1 antibody (^212^Pb-αVCAM-1) was assessed in comparison to conventional WBRT (27). In this preclinical study, BM were induced by intracardiac injection of human breast cancer cells (MDA-231-Br). ^212^Pb-αVCAM-1 showed a favourable biodistribution in the whole body with a high uptake in BM compared to healthy tissues. In addition, low toxicity was observed, highlighting the added value of ^212^Pb-αVCAM-1 in comparison to WBRT with respect to the avoidance of dose deposition in healthy brain. In terms of tumour control, tumour volume and the number of BM were both decreased in comparison to the WBRT group, and the OS was significantly increased (**Figure 1**). To understand the different therapeutic effects of ^212^Pb-αVCAM-1 and WBRT, clonogenic assays were performed and showed higher radiosensitivity parameters, such as the survival fraction at 2 Gy or the dose that decreased the survival fraction by 50% (28), for ^212^Pb-αVCAM-1 compared to WBRT. However, such an evaluation of radiosensitivity requires complex experiments with clonogenic assays that are not possible in clinical practice. Thus, evaluation of the added value of targeted RIT, in comparison to conventional WBRT for the treatment of BM, requires a detailed dosimetry as performed for external beam RT.

### Dosimetry of Radioimmunotherapy

Administered activity is either based on a fixed amount or adjusted taking the body weight or body surface area of the patient into consideration and not planned to maximise the tumour dose, whilst sparing the organs at risk. Nevertheless, a substantial effort is being made to individualise patient treatments and to improve the accuracy of dosimetry procedures in the clinic. Important initiatives to standardise dosimetry include the internal dosimetry task force linked to EANM (29, 30), the Medical Internal Radiation Dose committee (31), or the EU consortium MEDIRAD (32, 33). Dosimetry should play an important role when a new agent for RIT undergoes clinical testing, alongside the assessment of the maximum tolerated dose and side effects, similar to clinical trials of nonradioactive oncological drugs (34).

In the case of BM, only one study has reported dosimetry for the purpose of RIT. Poli and colleagues evaluated the biodistribution of ^124^I-L19SIP in patients with BM to compute the ^131^I-L19SIP dosimetry (8) With ^124^I, PET imaging can be performed, providing a more precise spatial information than SPECT, and its half-life of 4.18 days is compatible with a biodistribution study of ^131^I-L19SIP. PET imaging was performed at 1, 2, 24, 48, and 96 hours after an injected activity of (^124^I-L19SIP). Time activity curves were obtained and the cumulated activity over time (AUC) was computed. Using OLINDA/EXM (35) and MIRD formalism, the authors evaluated the dose in Gy per injected MBq in different organs. This study suggested that immuno-PET diagnostic imaging with ^124^I-L19SIP could actually predict the dose that would be delivered to the tumour *vs.* healthy organs by a subsequent therapeutic ^131^I-L19SIP dose. Those authors also measured the red bone marrow dose. The estimated dose was similar during both the diagnostic scanning and treatment. However, significant heterogeneity in the dose delivered to different tumours in the same patient was apparent. This tumour heterogeneity highlights the importance of preliminary dosimetry before RIT (8).

The biological distribution of ^212^Pb-αVCAM-1 has not yet been studied in humans. Two approaches to dosimetry can be considered, either during or before treatment. Concerning the first approach, the temporal and spatial distribution of ^212^Pb-αVCAM-1 can be evaluated through the γ-emissions of ^212^Pb with multiple time-point SPECT acquisitions. The second approach, similar to Poli et al. consists of performing pre-treatment imaging with ^203^Pb-αVCAM-1 (36), emitting γ-rays with a half-life of 51.87 h, to compute a predictive dose distribution of ^212^Pb-αVCAM-1. As the physical half-life of ^212^Pb is shorter (10.4 h) than that of ^131^I-L19SIP (37), all of the imaging acquisitions and blood sampling could be performed within one day instead of four. This imaging procedure would enable a dose computation to be made before treatment and, consequently, allow the possibility of adapting the injected activity to reach the desired dose to the tumour whilst minimising the risk of complication. Additionally, the injected activity or number of cycles could be tailored to reach the best TCP, whilst lowering the NTCP as in WBRT. For instance, the TCP model of WBRT is based on a biologically effective dose of about 40 Gy (38). Usually, a fractionation of 10 times 3 Gy is delivered to the patient after a CT planning scan. Calculation of the absorbed dose is relatively straightforward in EBRT with constant dose rates and photon beams of widely used energies, and the biological effects are mainly produced by low LET particles. It is hazardous to extrapolate doses from EBRT to RIT owing to the fundamental differences in the dose rate and the mechanisms of DNA damage and repair. Consequently, preclinical studies to characterise the biodistribution of ^212^Pb-αVCAM-1 with ^203^Pb-αVCAM-1 in BM is the next step to determining a predictive dose.

### Radioimmunotherapy of Late Stage BM

Most patients are treated when their BM are advanced due to the diagnostic insensitivity of the currently available imaging. Advanced BM are associated with larger tumour sizes and the microenvironment is different with marked angiogenesis suggesting BBB disruption, chaotic and heterogeneous vascularisation. Hypoxic features have been shown in the BM microenvironment from lung, breast, renal, and colorectal cancers (39), as well as at the preclinical level for BM from lung cancer (40). All of these changes negatively affect the performance of RIT, and fewer abnormal vessels (per tumour volume) is likely to reduce the accumulation of RIT at the tumour site. Poor concentration of RIT limits irradiation of the tumour by short range α-emitting. Imaging studies prior to RIT are, therefore, very important to evaluate biodistribution. However, RIT using α-emitting isotopes could still be very effective in combination with external RT for late BM, because α-particle radiation effects are not impacted by radioresistance factors such as hypoxia. The maximum relative radiosensitivity of cells to oxygen concentration, or the oxygen enhancement ratio (OER), is commonly thought to be about 3 for x-rays that induce secondary electrons with LET of 1.3 keV/µm. For α-particles with LET of 60–110 keV/µm, the OER decreases to 1.3–2.1 between the particle emission point and the Bragg peak, and to about 1.0 in the Bragg peak area, and thus leads to a no effect of hypoxia on RIT efficacy (41). However, this potential of the α-particle RIT for the treatment of hypoxic BM has first to be confirmed in preclinical studies, and then in clinical studies.

## Conclusion

Treatment of BM at the early stage of development is likely to yield optimal tumour control. However, owing to the small size of BM at this early stage, and limited detection, external RT is not suitable and molecularly targeted treatment is needed. RIT using α-particles (e.g., ^212^Pb) combined with biomarkers of early disease, such as cell adhesion molecules (e.g., VCAM-1), has shown promising results at the preclinical level for treatment of early BM. The treatment of well-established BM exhibiting hypoxic features with RIT using a combination of α-particles with a hypoxia biomarker may have a potential, but requires validation. Finally, because α-particles have a very high LET and a very short range, the distribution of α-particle RIT to the BM site is crucial. Consequently, imaging enabling the characterisation of biodistribution and dosimetry are needed to fully evaluate the potential benefit of α-particle RIT for the treatment of BM.

## Author Contributions

Manuscript drafting or manuscript revision for important intellectual content: all authors. All authors contributed to the article and approved the submitted version. Literature research: AC-D, CJ, NF, A-MF. Manuscript editing: All authors. Figure preparation: AC-D.

## Funding

This study was funded by the Région Normandie, the Centre National de la Recherche Scientifique (CNRS), the Université de Caen Normandie (UNICAEN), the European Union-Fonds Européen de Développement Régional (FEDER), the Ministère de l’Enseignement Supérieur et de la Recherche and the French National Agency for Research “Investissements d’Avenir” n° ANR-11-LABEX-0018-01 and n°ANR-10-EQPX1401 and The HABIONOR European project, and co-funded by the Normandy County Council, the French State in the framework of the interregional development Contract “Vallée de la Seine” 2015-2020 and the FRC. NS and KV were supported by a Cancer Research UK core award (C5255/A15935). 

## Conflict of Interest

Author NF was employed by GenesisCare.

The remaining authors declare that the research was conducted in the absence of any commercial or financial relationships that could be construed as a potential conflict of interest.

## Publisher’s Note

All claims expressed in this article are solely those of the authors and do not necessarily represent those of their affiliated organizations, or those of the publisher, the editors and the reviewers. Any product that may be evaluated in this article, or claim that may be made by its manufacturer, is not guaranteed or endorsed by the publisher.

## Abbreviations

BM, brain metastases; RIT, radio-immunotherapy; TCP, tumor control probability; NTCP, normal tissue complication probability; MRI, magnetic resonance imaging; PET, positron emission tomography; SPECT, single photon emission computed tomography; TKI, tyrosine kinase inhibitors; BBB, blood-brain-barrier; WBRT, whole brain radiotherapy; SF, survival fraction; MIRD, medical internal radiation Dose; EBRT, external beam radiotherapy; LET, linear energy transfer.
